# The influence of sex on activity in voluntary wheel running, forced treadmill running, and open field testing

**DOI:** 10.21203/rs.3.rs-4365992/v1

**Published:** 2024-05-14

**Authors:** Adam J. Janowski, Giovanni Berardi, Kazuhiro Hayashi, Ashley N. Plumb, Joe B. Lesnak, Tahsin Khataei, Ben Martin, Christopher J. Benson, Kathleen A. Sluka

**Affiliations:** University of Iowa; University of Iowa; University of Iowa; University of Iowa; University of Texas at Dallas; University of Iowa; University of Iowa; University of Iowa; University of Iowa

## Abstract

**Introduction:**

Physical activity is commonly used for both measuring and treating dysfunction. While preclinical work has been historically biased towards males, the use of both male and female animals is gaining popularity after multiple NIH initiatives. With increasing inclusion of both sexes, it has become imperative to determine sex differences in common behavioral assays. The purpose of this study was to determine baseline sex differences in 3 activity assays: voluntary wheel running, forced treadmill running, and open field testing.

**Methods:**

This was a secondary analysis of sex differences in healthy mice in 3 different assays: Separate mice were used for each assay. Specifically, 16 mice underwent 28 days of voluntary wheel running, 178 mice underwent forced treadmill running, and 88 mice underwent open field testing. Differences between sex across several activity parameters were examined for each assay.

**Results:**

In voluntary wheel running, sex differences with larger effect sizes were observed in distance run, running time, and bout duration, with smaller effect size differences in speed, and no difference in total bouts. In forced treadmill running, differences were shown in time to exhaustion, but no difference in max speed attained. In open field, there were sex differences in active time but not in distance and speed in data aggregated over 30 minutes; however, distance and speed in male mice showed a downward trajectory over the final 20 minutes of testing, whereas females maintained the same trajectory.

**Conclusion:**

These data suggest that male mice demonstrate comparable activity intensity as female mice but do not match female’s duration of activity, especially for volitional tasks. Researchers utilizing these assays should account for sex differences as they could potentially mask true findings in an experiment.

**Plain English Summary:**

Physical activity is a common measure to examine function in human subjects with and without disease. Animal models often use measures of physical activity to assess function, yet most of these measures have been done in males only, making interpretation and translation to females and humans difficult. Several measures have been used to measure activity in animals, including those examining voluntary running behavior, maximum capacity, and general activity levels; sex differences between these measures are unclear. We discovered sex differences throughout each of three activity tests. In voluntary running behavior there were large differences between sexes with females running a greater distance and spending more time running. There were small differences in the maximum capacity with females running for a longer period at high intensity. General activity levels showed small differences with females being less active than males. Thus, the greatest differences were found for voluntary running and small differences were found for maximum capacity and general activity levels; differences observed were dependent on the task. Researchers utilizing these assays should account for sex differences as they could potentially mask true findings in an experiment.

## Background

The World Health Organization defines physical activity as any type of bodily movement produced by skeletal muscles that requires the expenditure of energy [1]. This broad reaching definition includes activities as small as short periods of standing or walking, all the way to the intense exercise performed by elite athletes. Physical activity is effective in the primary and secondary prevention of chronic diseases [2–4] and elicits changes in multiple biological systems [5–9]. Physical activity levels can also be used as a test of functional ability allowing researchers to determine differences in physical ability between populations, as well as change in activity due to specific interventions [10–12].

Sex-specific differences in physical activity and performance-based tests in human subjects are well known, with females generally demonstrating reduced fatigability compared to male counterparts [13–17]. Historically there has existed a strong sex bias in preclinical research, evidenced by more frequent utilization of male animals relative to females. As inclusion of both sexes in preclinical research becomes more common, it is important to determine inherent sex differences in common preclinical behavioral assays. Greater resolution of the sex-specific differences in rodents will allow parallel studies and better translation between humans and animals.

Three commonly utilized activity assays in rodents are voluntary wheel running, forced treadmill running, and open field testing, and are used to measure the impact of pain, fatigue, motivation, and psychological states [18–22]. Voluntary wheel running assesses self-selected activity in a stress-free home environment removed from tester influence [23]; forced treadmill running to exhaustion assesses maximal exercise performance [21, 24]; and open field testing assesses self-selected exploratory behavior in a novel environment. The results obtained from each are uniquely meaningful. Prior work shows female animals run a greater distance and spend more time on a running wheel than males. However, the data regarding sex-specific differences in speed, bouts, and duration in running wheels exhibit mixed results, while forced treadmill running and open field testing show multiple outcomes for various measures [21, 25–36]. Thus, a better understanding of sex-specific differences in activity measures are needed.

Advances in automated data collection and analysis allow examination of multiple indices of physical activity that may provide greater insight into different aspects of activity and function, thereby allowing for greater translation from preclinical to clinical work [23, 25]. For example, individuals with chronic pain show decreased total activity and lower peak activity with use of activity monitors (voluntary physical activity), but demonstrate similar functional capacity with controlled performance-based tests [37, 38]. Activity measures in animals are designed to assess motivational, cognitive, and emotional states [39–41] making detailed delineation of sex differences among these assays important for study design, analysis, and interpretation. Therefore, the purpose of this study was to examine sex differences in three common activity-based measures: voluntary wheel running, forced treadmill running, and open field exploratory behavior.

## Materials and Methods

**Figure 1:**
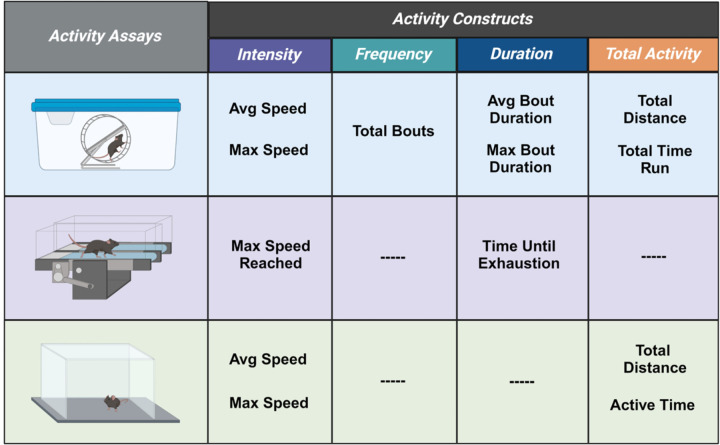
Schematic diagram showing three different activity assays. Three activity assays were utilized to investigate sex differences: voluntary wheel running, forced treadmill running, and open field testing. Assays were broken down into different activity constructs to analyze differences with greater granularity. Graphic designed in Biorender.

This study was a secondary analysis of data that was accumulated throughout multiple experiments. Each assay was the first test performed on the individual animals as part of different experimental protocols and was performed prior to any drug or behavioral intervention. Three assays were utilized including voluntary wheel running, forced treadmill running, and open field (Fig. 1). Baseline performance measures were utilized in the case of treadmill running and open field testing, while 4 weeks of free wheel access were utilized for voluntary wheel running. Male (n = 122) and female (n = 166) mice utilized in all experiments were C57BL/6J aged 8–11 weeks (Jackson Laboratories, Bar Harbor, ME, USA). Separate animals were used for each assay. All mice were housed on a 12-hour light–dark cycle with access to food and water ad libitum. All experiments were approved by the University of Iowa Animal Care and Use Committee and were conducted in accordance with the National Institute of Health’s Guidelines for the Care and Use of Laboratory Animals.

### Voluntary Wheel Running

Voluntary wheel running was tested using 32 mice (16 male and 16 female). Mice were run in 4 separate cohorts of 8 (4M/4F) at a time. Mice were individually housed on the first day of testing and running wheels were immediately placed in each home cage (Columbus Instruments 0294–4019). No formal acclimation period was provided. Running wheel activity was recorded for 28 days. On day 14, home cages and running wheels were changed. Revolutions were compiled to a spreadsheet every minute using Columbus Instrument Windows software. Because mice run primarily during the night cycle, only the 12-hour night cycle running period was used in analysis [22, 23]. Data output was analyzed with a custom python script which calculated total distance run per day (distance), percent of total minutes where running occurred (total time run), average wheel revolutions during minutes where running occurred (average speed), and the maximum revolutions in one minute (max speed). The total number of running bouts, average duration of each running bout, and the duration of the longest running bout were also calculated. A bout of running was defined as a period of consecutive minutes where any amount of running occurred. If running stopped long enough for 1 recorded minute to pass, then a bout was considered terminated. The average bout duration was determined as the average length of each bout and the max bout duration was the longest recorded bout. All measures of wheel revolutions were converted to distance in meters for further analysis.

### Treadmill

Maximal treadmill running duration was tested utilizing 168 mice (62 male and 106 female). The treadmill protocol has been described previously [24]. Briefly, mice underwent a 5-day acclimation period on the treadmill (Columbus Instruments) for 30 minutes daily with gradual increases in speed and incline. Electrical shock grids (1 mA, 1 Hz, 200 ms duration) were present on the rear of treadmill to motivate maximal ambulatory time and speed during acclimation days. On testing day, the treadmill was set to a 20-degree incline and shock grids were turned off. Mice were given a 10-minute period to acclimate to the treadmill after which they were given a 10-minute warmup at 6 m/min. Speed was then increased to 8 m/min for 3 minutes and subsequently increased 2 m/min every 3 minutes until the mouse reached exhaustion. Shock bars remained off during testing, but gentle prodding was used with a tongue depressor to encourage running. Exhaustion was determined as the point where mice resisted prodding for 10 consecutive seconds. Total running time was recorded and analyzed. Additionally, maximum speed attained by the animal was analyzed using a subset of animals (32M, 82F) for whom that data was documented.

### Open Field

Open field testing was performed in 88 mice (44 male and 44 female). Mice were placed in one of four 16-inch X 16-inch open-topped boxes with translucent walls. Solid barriers were present between cages so mice could not see each other. Recordings were completed without an investigator in the room. All testing was performed between 11 AM and 2 PM to minimize the impact of time-of-day on data collection. Lumen level was consistent at ~ 140 lux during testing and throughout the middle and corners of all individual boxes. Box floors were cleaned thoroughly between each bout of testing.

Activity in the open field was recorded over 30 minutes at one-second intervals using an overhead camera (Panasonic WV-BP334) and Limelight tracking and analysis software (Version 2.7). The 30-minute testing period was evaluated in total as well as in 5-minute intervals using a custom python script to determine distance run, active time, average speed, and max speed. Max speed was determined as the average of the top 10 fastest seconds run. Additionally, the limelight software assessed time spent in the middle 50% area of the open field box.

### Statistical Analysis

Voluntary running wheel data was analyzed using linear mixed effects models. The primary outcomes (i.e., distance, time run, etc.) were modeled as a function of sex, time, and sex*time interaction. Two unique models were constructed for each measurement, the first model included days 1–14 as time points and the second model included days 15–28. Previous research and unpublished data from our laboratory suggest that mice require roughly two weeks to reach a normalized daily running distance, but with noted variability [25–28]. Therefore, the first model (days 1–14) was arranged specifically to measure sex differences in day 1 running as well as the rate of change (slope) in running over the first 14 days. The second model (days 15–28) was analyzed similarly, examining sex differences in wheel running on day 15 and the rate of change from days 15–28; days 15–28 were after habituation had occurred. Each model was tested for the inclusion of random slopes and random intercepts. If the addition of random slopes proved statistically significant via partial F-test then random slopes and intercepts were utilized. Otherwise, only random intercepts were utilized. Model fit was validated using QQ plots and residual plots.

Voluntary wheel running was also analyzed by calculating the area under the curve (AUC) over the full 28-day period. Voluntary wheel running AUC along with forced treadmill running, and open field data were first tested for normal distribution using the Shapiro-Wilks test. T-tests and Cohen’s D effect sizes were used to analyze sex differences in voluntary wheel running AUC, forced treadmill running (time to exhaustion and max speed), and open field testing (total distance run, active time, average speed, and max speed over 30 mins). Open field data was segmented into 5-minute intervals for time-based longitudinal analysis. These changes in open field distance, active time, average speed, and max speed over time were analyzed using linear mixed effects models. The analysis involved splitting the measurements into the first 10 minutes and final 20 minutes, using separate models to determine initial running differences in main effects and interactions as well as trends throughout the assay akin to voluntary wheel running analysis above. To account for multiple comparisons, statistical tests for each assay were adjusted using the Benjamini-Hochberg method to control the false discovery rate [42]. This adjusted value is presented as a q-value. Both q-values and p-values are presented in Tables 1–3. Only q-values are presented in the text of the article and a q < 0.05 is considered significant.

## Results

### Voluntary Wheel Running Task

#### Total Activity Volume: Total distance and total time run

##### Distance.

**Figure 2:**
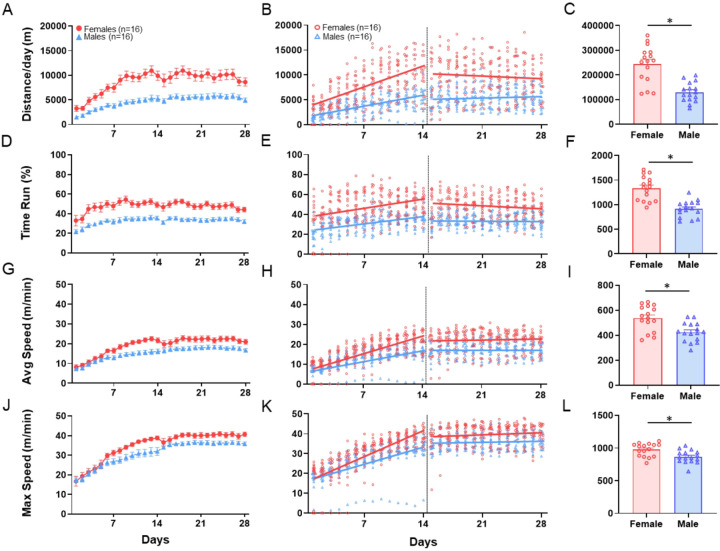
Running wheel behavior – distance, time, and speed. Results from voluntary wheel running analysis of distance (**A-C**), total time run (**D-F**), average speed (**G-I**) and max speed (**J-L**). Graphs in the left column (**A, D, G, J**) show daily data over the 28-day time period for male and female mice. Data are represented as the mean + S.E.M. Graphs in the middle column (**B, E, H, K**) show individual data points for each animal and the regression lines for the acclimation phase, Days 1–14, and for the plateau phase, Days 15–28. The right column (**C, F, I, L**) shows summary bar graphs for the AUC for the entire 28-day period. Data are represented as the mean + S.E.M. *, q<0.05.

Voluntary wheel running was recorded only during the 12-hour dark cycle. On the first day in running wheels, females ran an average distance of 3158 m compared to males who ran 1699 m (sex effect: β = 1459, q = 0.034) (Fig. 2A & 2B, Table 1). Over the first 14 days, both males and females continued to increase their running distance with females increasing their distance at a greater rate; females increased at 501 m/day and males 281 m/day (time effect: β = 281, q < 0.001; sex*time: β = 320, q < 0.002) (Fig. 2A & 2B, Table 1). During days 15–28, females ran an average distance of 11,062 m/day greater than double the distance compared to males at 5221 m/day (sex effect: β = 5842, q < 0.009) (Fig. 2A & 2B, Table 1). AUC over the entire 28-day period showed that females (243 km ± 77) ran a greater distance than males (134 km ± 37) (q < 0.001, δ = 1.8) (Fig. 2C, Table 2).

##### Time.

On day 1, females ran an average of 39% of the time, whereas males ran an average of 25% of the time (sex effect: β = 14.35, p = 0.009) (Fig. 2D & 2E, Table 1). Both males and females increased total run time by ~ 1% per day over 14 days with no difference in the rate of increase. Days 15–28, females ran on average 56% of the time and males 33% of the time (sex effect: β = 22.17, q < 0.001) (Fig. 2D & 2E, Table 1). AUC over the entire 28-day period showed that females (1329 ± 253) ran a greater percent of the time than males (929 ± 157) (q < 0.001, δ = 1.80) (Fig. 2F, Table 2).

#### Activity Intensity: Average and Peak Speed

As a measure of speed, we quantified both the average and max speed in a one-minute period (m/min) daily, over the entire 28-days. On day 1 in running wheels there was no difference between males and females in average speed (sex effect: β = 0.12, q = 0.95) (Fig. 2G & 2H, Table 1) or max speed (sex effect: β=−0.58, q = 0.86) (Fig. 2J & 2K, Table 1). During the first 14 days, males increased average speed daily by 0.68 m/min (time effect: β = 0.68, q < 0.001) (Fig. 2G & 2H, Table 1) and females by 1.17 m/min each day (sex*time: β = 0.49, q = 0.004) (Fig. 2G & 2H, Table 1). Males increased max speed an average of 1.18 m/min each day (time effect: β = 1.18, q < 0.001) (Fig. 2J & 2K, Table 1) and females 1.76 m/min (sex*time: β = 0.58, q = 0.019) (Fig. 2J & 2K, Table 1). During days 15–28, males and females achieved daily average speeds of 16.7 and 20.4 m/min respectively (sex effect: β = 3.70, q = 0.35) (Fig. 2G & 2H, Table 1) and max speeds of 34.2 and 35.7 m/min (sex effect: β = 1.48, q = 0.71) (Fig. 2J & 2K, Table 1). These differences were not statistically significant. However, AUC over the entire 28-day period showed that females ran greater average (539 ± 105) and max (982 ± 99) speed than males (432 ± 70) (872 ± 99) (average: q < 0.011, δ = 1.19) (max: q < 0.015, δ = 1.11) (Fig. 1I & 1J, Table 2).

#### Frequency and Duration of Activity: Bout Number and Duration

**Figure 3:**
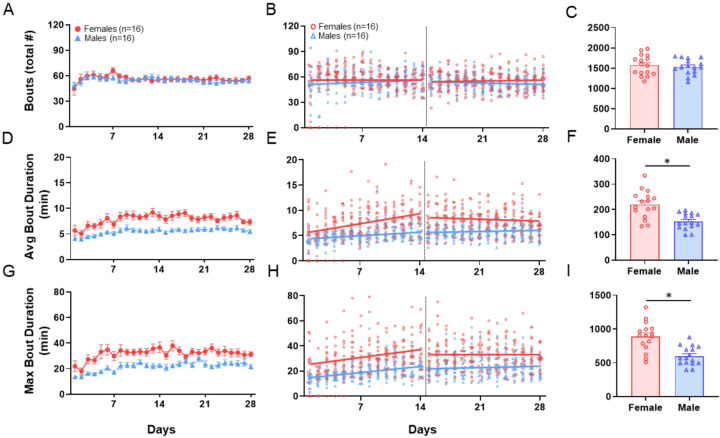
Running Wheel Behavior – Bouts. Results from voluntary wheel running analysis of total bouts (**A-C**), average bout time (**D-F**), and max bout time (**G-I**). Graphs in the left column (**A, D, G**) show daily data over the 28-day time period for male and female mice. Data are represented as the mean + S.E.M. Graphs in the middle column (**B, E, H**) show individual data points for each animal and the regression lines for the acclimation phase, Days 1–14, and for the plateau phase, Days 15–28. The right column (**C, F, I**) shows summary bar graphs for the AUC for the entire 28-day period. Data are represented as the mean + S.E.M. *, q<0.05.

We examined bouts run (total number), average bout duration (in minutes), and max bout duration (in minutes) as measures of frequency and duration of activity. On day 1 in running wheels there was no sex difference in total bouts run (sex effect: β = 2.85, q = 0.77) (Fig. 3A & 3B, Table 1) or average bout duration (sex effect: β = 1.41, q = 0.083) (Fig. 3D & 3E, Table 1); however, females had a longer max bout duration (sex effect: β = 10.32, q = 0.015) (Fig. 3G & 3H, Table 1) with an average of 23.9 minutes compared to males who averaged 13.6 minutes. During days 1–14 there was no difference in the rate of increase of bouts run (sex*time: β=−0.24, q = 0.78) (Fig. 3A & 3B, Table 1), average bout duration (sex*time: β = 0.13, q = 0.12) (Fig. 3D & 3E, Table 1), or max bout duration (sex*time: β = 0.15, q = 0.78) (Fig. 3G & 3H, Table 1) between sexes. During days 15–28 females ran 56.8 total bouts and males 58.1 total bouts on average, which was not a statistically significant difference (sex effect: β=−1.37, q = 0.83) (Fig. 3A & 3B, Table 1). Also, during days 15–28, female’s average daily bout duration was 9.6 minutes compared to 5.3 minutes for males (sex effect: β = 4.21, q = 0.008) (Fig. 3D & 3E, Table 1) and female’s daily max bout duration was 32.9 min compared to 20.8 minutes for males (sex effect: β = 12.11, q = 0.059) (Fig. 3G & 3H, Table 1). AUC over the entire 28-day period showed that females (1599 ± 235) ran the same number of bouts as males (1530 ± 199) (q = 0.56, δ = 0.32) (Fig. 3C, Table 2), while it showed that females had a greater average (216 ± 55) and max (884 ± 223) bout duration than males (157 ± 29) (619 ± 144) (average: q = 0.006, δ = 1.36) (max: q = 0.004, δ = 1.41) (Fig. 3F & 3I, Table 2).

### Forced Treadmill Running (Maximum Exercise Capacity)

**Figure 4:**
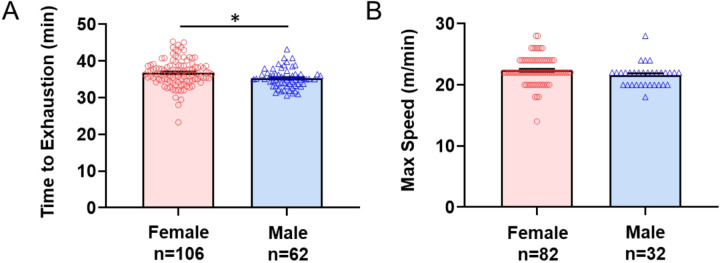
Forced Treadmill Running. Bar graphs and individual data points for male and female mice for **A)** time to exhaustion and **B)** maximum speed of running reached. Data are represented as the mean + S.E.M. q<0.05.

To examine sex differences in maximal capacity, we used a forced treadmill task that increased belt speed until exhaustion. During the forced treadmill task, males ran an average of 35.3 min and females ran 36.8 min, which was significantly different (p = 0.004, δ = 0.46) (Fig. 4A, Table 2). The maximum speed attained during the exercise protocol was no different between males (21.60 ± 1.81 m/min) and females (22.40 ± 2.27 m/min) (p = 0.052, δ = 0.37) (Fig. 4B, Table 2). Thus, females had a longer time till exhaustion compared with males, but sexes did not differ in maximum speed attained.

### Open Field Testing: Spontaneous Exploratory Behavior in a Novel Environment

#### Distance.

**Figure 5:**
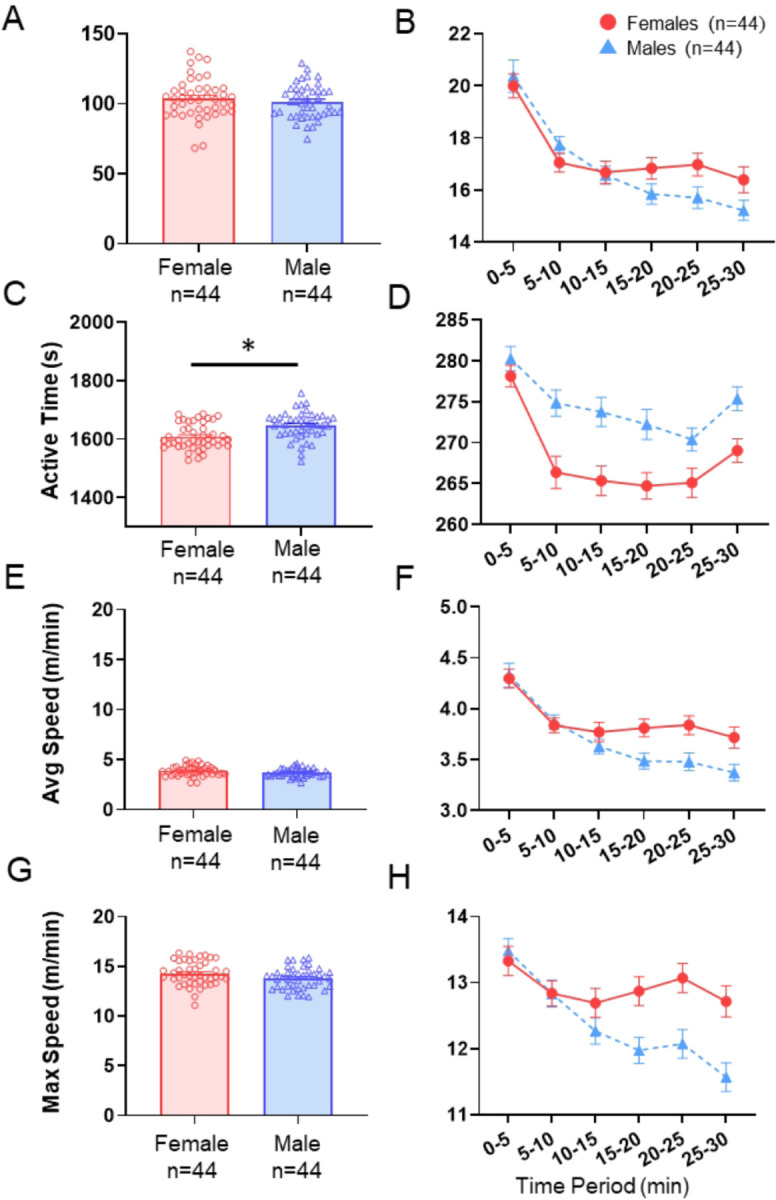
Open Field Test. **A.** Bar graphs and individual data points for total distance traveled during the 30-minute period for female and male mice. **B.** Line graph showing distance run over the 30-minute recording session broken into 5-minute intervals. **C.** Bar graphs and individual data points for time spent moving during the 30-minute period for female and male mice. **D.** Line graph showing time spent moving over the 30-minute recording session broken into 5-minute intervals of time. **E.** Bar graphs and individual data points for average speed over the 30-minute period while moving for female and male mice. **F.** Line graph showing average speed over the 30-minute recording session broken into 5-minute intervals of time. **G.** Bar graphs and individual data points for maximum speed achieved during the 30-minute period for female and male mice. **H.** Line graph showing maximum speed achieved in each of 5-minute intervals over the 30-min period. Data are the mean + S.E.M. *, q<0.05

Over the course of 30 minutes in the open field, males ran a similar average distance (101.43 m ± 12.13) compared to females (103.95 m ± 14.51) (q = 0.52, δ = 0.19) (Fig. 5A, Table 2). When examining 5-minute segments, both sexes equally decreased distance in the first 10-minutes (time effect: β=−2.64, q < 0.001) (sex*time: β=−0.31, q = 0.74) (Fig. 5B, Table 3). However, in the final 20-minutes the distance run by male mice steadily decreased in distance (time effect: β=−0.59, q < 0.001) relative to females (sex*time: β = 0.49, q = 0.002) (Fig. 5B, Table 3).

#### Active Time.

Over the course of 30 minutes, males demonstrated more active time in the open field (1646.75 s ± 43.56) on average compared to females (1608.55 s ± 47.03) (q < 0.001, δ = 0.84) (Fig. 5C, Table 2). When breaking down active time into 5-minute segments, both sexes decreased active time over the first 10 minutes (time effect: β=−5.41, q = 0.009) (Fig. 5D, Table 3) with females decreasing greater than males (sex*time: β=−6.36, q = 0.034) (Fig. 5D, Table 3). Over the final 20 minutes in the open field, males had more active time than females (sex effect: β=−10.16, q = 0.008) (Fig. 5D, Table 3) but with no significant time effect or sex*time interaction (time effect: β=−0.23, q = 0.73) (sex*time effect: β = 0.74, q = 0.35) (Fig. 5D, Table 3).

#### Average Speed.

Males and females demonstrated no difference in average speed throughout 30 minutes with males running on average 3.69 m/min and females 3.88 m/min (q = 0.13, δ = 0.39) (Fig. 3E, Table 2). When breaking down the 5-minute segments of running, both sexes decreased equally over the first 10 minutes in the open field (time effect: β=−0.46, q < 0.001) (sex*time: β = 0.00, q = 0.99) (Fig. 5F, Table 3). Over the final 20 minutes, males had a greater decrease in average running speed compared to females (time effect: β=−0.11, q < 0.001) (sex*time: β = 0.10, q = 0.004) (Fig. 5F, Table 3).

#### Maximum (Max) Speed.

Over the course of 30 minutes males ran at a similar max speed (13.80 m/min ± 1.06) compared to females (14.29 m/min ± 1.21) (q = 0.087, δ = 0.43) (Fig. 5G, Table 2). When examining 5-minute segments, both sexes decreased running equally over the first 10 minutes in the open field (time effect: β=−0.65, q < 0.008) (sex*time: β = 0.16, q = 0.73) (Fig. 5H, Table 3). Over the final 20 minutes, males had a greater decrease in max running speed compared to females (time effect: β=−0.27, q < 0.001) (sex*time: β = 0.29, q .2 0.001) (Fig. 5F, Table 3).

#### Time in Center.

Over the course of 30 minutes in the open field males spent a similar amount of time in the center of the testing box (14.5 m ± 4.3) compared to females (15.2% ± 4.8) (q = 0.74, δ = 0.14) (Table 2).

## Discussion

The current study shows that there are sex differences in outcome measures for these commonly used assays. Specifically, we show voluntary wheel running, females ran greater distance and total time than males, consistent with prior studies [22, 43, 44]. Uniquely, we show that males and females initiate running bouts an equal number of times, but both average and max bout duration is greater in females, which likely contributes to greater distance and time run observed in females. In forced treadmill running, females ran for a significantly longer period, but obtained the same top speed as males. In open field testing there were no sex differences in total distance and speed, but there were sex differences in active time. Thus, each assay appears be a unique measure of activity with voluntary wheel running showing the largest sex-specific differences. Importantly, if these assays are being utilized to test new drugs or animal models researchers should consider accounting for sex differences as they could potentially mask the true findings in an experiment.

### Voluntary Wheel Running

One of the strengths of voluntary wheel running is that it is a measure of self-initiated activity in a non-stressful environment [25, 45–47]. Activity is recorded during the dark phase without any human interaction and occurs during their naturally active period. The use of computer-based data collection allows for a more complete analysis of voluntary wheel running behavior and may correlate to physical activity levels in human subjects [48]. However, because wheel running is self-selected, there is no control or standardization of the task, and thus total activity varies between each individual animal.

Consideration of sex differences in wheel running behavior began in the early 1920’s and numerous data support prominent sex differences in rodents; specifically, females run a greater distance and total time than males [43, 44, 49]. We expanded these results by showing a similar number of bouts between sexes, with females showing greater bout duration, greater speed, and greater rate of increase to plateau, all of which contribute to greater distance run.

A bout is defined as a brief period of increased activity and has been considered an important component of physical activity [1, 4]. For example, the American College of Sports Medicine recommends individuals accumulate 150 minutes of moderate to vigorous activity per week with bout durations of at least 10 minutes. However, clinicians often recommend increasing physical activity levels regardless of bout duration [50, 51] and prior research suggests improvements in function and pain, regardless of bout duration [52, 53]. The current study showed a similar number of bouts per day between sexes, but longer bout duration in females. Data were captured in 1-minute intervals, and bouts were separated by at least one minute without running wheel activity. Conversely, De Bono et al. showed females ran a greater total number of bouts but showed no sex differences in bout duration – data were collected in 5-second intervals, but it is unclear precisely how bouts were determined. This difference is likely related to how bouts were collected and calculated and could represent the difference between a technical bout and a biologically meaningful bout of activity. Prior work in humans have shown that bouts of < 10 minutes are associated with reduced frailty in both sexes [52], but activity intensity is a greater determinant of cardiometabolic risk than bout duration [54]. Total activity time regardless of bouts is related to fatigue, function, and disease severity [37], while the total minutes spent in ≥ 10-min bouts is associated with lower pain [55] in individuals with fibromyalgia, suggesting both total activity and bouts are important in clinical populations. Future work in preclinical studies is needed to determine meaningful bout-lengths.

The current study considered two phases of running wheel behavior; an acclimation phase during which animals increase their running distance each day, and a plateau phase where daily running distance has normalized. The current study showed that both male and female mice reached a plateau after 2 weeks for daily distance but varied for other measures. Total running time normalized after 5 days, total bouts after 3 days, and average and peak bout duration between 8–14 days. These data are consistent with prior studies who showed that speed of wheel running peaked within the 2 or 3 weeks in both sexes [26, 28], but contrast others showing shorter durations for acclimation and more prominent sex differences [25, 27]. Differences could be related to the type of running wheel used, the external environment, or age of the animals.

Longer-term acclimation is frequently performed to normalize wheel running. However, this is potentially problematic given the impact of voluntary wheel running, as a form of exercise, on physiological responses in multiple systems including body composition, muscular system, metabolic capacity, peripheral and central nervous system, and immune system [8, 22, 46, 56–61]. Physiological changes begin immediately with exercise and can produce biological effects within days [9, 46, 60, 62]. For example, running wheel activity in mice can prevent the development of chronic muscle pain and associated changes in the central nervous system with just 5 days of activity [60]. Therefore, if experimental mice undergo 2–3 weeks of wheel running to normalize running distance, it is likely these mice undergo significant physiological adaptations that alter the responses observed in otherwise sedentary mice.

### Forced Treadmill Running

Forced treadmill running is utilized to determine maximal exercise capacity and allows for control of speed and intensity. To maintain running, electric shock is often used to maintain running and can cause stress [21, 24]. The current study showed females ran 1.5 minutes, 4.3%, longer than males. This difference was statistically significant but whether the difference is biologically meaningful is debatable. As a secondary analysis we included the animals that were available, but for perspective, at an effect size of 0.46 and an alpha of 0.05 we needed an n = 75 to achieve 0.8 power. In contrast to our data, prior studies report female mice run 25–50% longer than males [31, 32] but female rats run 40% less than males [33]. These differences are likely related to differences in the protocols, species (rat vs. mice), different treadmill inclines (10 vs, 20-degrees), time spent at lower speeds, or rate of speed increase.

### Open Field Testing

The current study showed no differences in activity between sexes for open field testing parameters of distance and speed, but there were significant differences in active time. These data generally agree with prior studies in C57BL/6J mice who show equivalent activity between sexes [29, 35, 36]. On the other hand, other mouse strains, MOLF and SJL, show higher activity in females [34]. Complicating comparison between studies is differences in individual testing parameters, including size of the open field chamber, lighting, and the transparency of the walls (clear versus opaque). While activity assessment of the 30-minute data in aggregate yielded no meaningful differences in distance and speed, a more in-depth analysis examining data in 5-minute segments revealed unique-sex-specific differences. During the final 20 minutes of the test females maintained running distance and speed similar to the first 10 minutes, while males showed steady decline during the last 20 minutes. This is a small but noteworthy effect, as it parallels the pattern of decreased duration of running in males that we see in voluntary wheel running.

### Mechanisms of sex differences in activity assays

Sexually dimorphic differences in activity might be due to variation in sex hormones, muscle capacity, or age. Multiple studies show increases in estradiol enhance wheel running activity in male and female mice [32, 63–67], but not exploratory behavior in the open field test [36, 68, 69]. Further, the sex-differences in total distance, total duration and speed of wheel running behavior disappears by 6–9 months of age [47]. Female mice show higher mobilization and use of lipids within the skeletal muscle than males and have more type I and less type II muscle fibers, which together could contribute to greater endurance [31, 32]. Thus, the sex-specific differences likely involve multiple mechanisms across systems.

### Strengths and Limitations

This study was a secondary analysis of baseline data, and thus was not designed specifically to test sex differences. However, protocols were consistent across animals in each cohort and data was taken at baseline prior to any intervention. Separate mice were utilized for each assay; therefore, we were unable to correlate relationships within-mouse between the different activity assays. Similarly, we did not account for muscle volume or cross-sectional area. We collected wheel revolutions every minute. While common practice, extrapolating speed and bout data is less granular than previously reported by de Bono who collected every 5 seconds [25], and thus may be difficult to directly compare results between studies.

## Conclusions

Taken together, important patterns in sex differences emerge from our 3 assays. Females show a slightly greater activity intensity as evidenced by differences in speed (smaller effect sizes), similar frequency of activity evidenced by total bouts, and a robust difference in duration of activity evidenced by total distance (larger effect sizes). These differences were most pronounced for running wheel activity and did not translate to exploratory behavior in the open field test. Based on data from running wheel and treadmill tests, females appear to be less fatigable but have similar maximum ability to males. The results in mice parallel research in humans showing suggesting females are less fatigable [14–17] and engage in more frequent moderate and light exercise than males [70]. However, the parallels are not completely synchronous as human males demonstrate greater speed and power output [71].

## Figures and Tables

**Table 1:** Results from the voluntary wheel running linear mixed effects models.

	Days 1–14						Days15–28					
Variable	Effect	β	SE	t	p	q	Effect	β	SE	t	p	q
**Distance (m)**	*Intercept*	1698.94	398.58	4.26	< 0.001	---	Intercept	5220.76	1253.50	4.17	< 0.001	---
	*Sex*	1459.31	563.13	2.59	0.015	**0.034**	Sex	5842.21	1774.58	3.29	0.003	**0.009**
	*Time*	281.27	54.00	5.21	< 0.001	**< 0.001**	Time	13.31	40.04	0.33	0.742	0.826
	Sex*Time	320.33	76.20	4.20	< 0.001	**0.002**	Sex*Time	−70.77	56.73	−1.25	0.222	0.351
**Total Time Run (%)**	*Intercept*	24.46	3.11	7.88	< 0.001	---	Intercept	33.88	2.96	11.43	< 0.001	---
	*Sex*	14.35	4.39	3.27	0.003	**0.009**	Sex	22.17	4.20	5.28	< 0.001	**< 0.001**
	*Time*	0.94	0.25	3.75	0.001	**0.004**	Time	0.00	0.11	−0.01	0.992	0.992
	Sex*Time	0.19	0.35	0.54	0.596	0.733	Sex*Time	−0.36	0.15	−2.44	0.015	**0.034**
**Average Speed (m/min)**	*Intercept*	7.70	0.98	7.85	< 0.001	---	Intercept	16.72	2.05	8.18	< 0.001	---
	*Sex*	0.12	1.39	0.08	0.935	0.954	Sex	3.70	2.89	1.28	0.211	0.345
	*Time*	0.68	0.09	7.19	< 0.001	**< 0.001**	Time	0.04	0.07	0.59	0.557	0.718
	Sex*Time	0.49	0.13	3.68	0.001	**0.004**	Sex*Time	0.03	0.09	0.28	0.780	0.831
**Max Speed (m/min)**	*Intercept*	17.34	1.84	9.42	< 0.001	---	Intercept	34.16	1.74	19.63	< 0.001	---
	*Sex*	−0.58	2.60	−0.22	0.826	0.861	Sex	1.48	2.46	0.60	0.553	0.718
	*Time*	1.18	0.14	8.29	< 0.001	**< 0.001**	Time	0.08	0.06	1.39	0.176	0.309
	Sex*Time	0.58	0.20	2.88	0.007	**0.019**	Sex*Time	0.11	0.09	1.29	0.206	0.345
**Total Bouts**	*Intercept*	53.76	4.34	12.38	< 0.001	---	Intercept	58.13	3.17	18.35	< 0.001	---
	*Sex*	2.85	6.14	0.46	0.646	0.772	Sex	−1.37	4.49	−0.31	0.761	0.829
	*Time*	0.29	0.41	0.71	0.485	0.660	Time	−0.20	0.11	−1.90	0.059	0.111
	Sex*Time	−0.24	0.58	−0.41	0.684	0.779	Sex*Time	0.15	0.15	0.96	0.339	0.512
**Average Bout Time (min)**	*Intercept*	4.13	0.47	8.88	< 0.001	---	Intercept	5.34	0.88	6.06	< 0.001	---
	*Sex*	1.41	0.66	2.14	0.041	0.083	Sex	4.21	1.25	3.38	0.002	**0.008**
	*Time*	0.14	0.05	2.86	0.008	**0.019**	Time	0.02	0.03	0.71	0.485	0.660
	Sex*Time	0.13	0.07	1.89	0.068	0.124	Sex*Time	−0.09	0.04	−1.99	0.056	0.110
**Max Bout Time (min)**	*Intercept*	13.61	2.43	5.61	< 0.001	---	Intercept	20.77	3.86	5.39	< 0.001	---
	*Sex*	10.32	3.43	3.01	0.005	**0.015**	Sex	12.11	5.47	2.21	0.028	0.059
	*Time*	0.79	0.26	3.00	0.005	**0.015**	Time	0.14	0.15	0.95	0.345	0.512
	Sex*Time	0.15	0.37	0.42	0.680	0.779	Sex*Time	−0.11	0.22	−0.53	0.598	0.733

Each running wheel construct had two regressions performed: the first from day 1–14 and the second from day 15–28. Each model included a term for sex, time, and sex*time interaction. * SE, Standard Error; m, meters; min, minute; q, Benjamini-Hochberg multiple comparisons correction.

**Table 2:** Results from voluntary wheel running, forced treadmill running, and open field.

Test	Females (mean ± SD)	Males(mean ± SD)	p	q	d
**Running Wheels**					
Distance (m)	243414.5 ± 77327.0	134302.4 ± 36544.8	< 0.001	**< 0.001**	1.8
Percent Minute Run (AUC %)	1328.5 ± 253.9	928.9 ± 156.7	< 0.001	**< 0.001**	1.89
Average Meters/Minute (m)	538.5 ± 104.9	432.8 ± 70.0	0.003	**0.011**	1.19
Max Meters/Minute (m)	982.2 ± 98.8	872 ± 98.9	0.005	**0.015**	1.11
Running Bouts	1598.9 ± 235.3	1529.6 ± 198.8	0.391	0.563	0.32
Average Bout Time (min)	215.9 ± 54.5	156.7 ± 29.1	0.001	**0.006**	1.36
Max Bout Time (min)	883.9 ± 223.2	618.7 ± 143.8	< 0.001	**0.004**	1.41
**Treadmill**					
Running Duration (min)	36.79 ± 3.59	35.29 ± 2.58	0.002	**0.004**	0.46
Max Velocity Reached (m/min)	22.4 ± 2.27	21.6 ± 1.81	0.052	0.052	0.37
**Open Field**					
Distance (m)	103.95 ± 14.51	101.43 ± 12.13	0.379	0.523	0.19
Active Time (s)	1608.55 ± 47.03	1646.75 ± 43.56	< 0.001	**< 0.001**	0.84
Average Velocity (m/min)	3.88 ± 0.51	3.69 ± 0.42	0.073	0.133	0.39
Max Velocity (m/min)	14.29 ± 1.21	13.80 ± 1.06	0.045	0.087	0.43
Percent in Center (%)	15.2 ± 4.8	14.5 ± 4.3	0.664	0.741	0.14

Results include AUC values of the 7 different voluntary wheel running constructs, as well as forced treadmill running duration and max velocity, and results from the whole 30-min open field test. Sex differences were assessed using t-tests comparing males and females, adjustment for multiple comparisons (q), and effect sizes (Cohen’s D). Data presented as means ± SD. * SD, Standard deviation; m, meters; min, minute; s, seconds; d, Cohen’s D; AUC, area under curve.

**Table 3:** Results from the open field test linear mixed effects models.

	0–10minutes						10–30 minutes					
Variable	Effect	β	SE	t	p	q	Effect	β	SE	t	p	q
**Distance (m)**	*Intercept*	22.99	0.80	28.73	< 0.001	---	*Intercept*	18.56	0.45	41.62	< 0.001	---
	*Sex*	−0.06	1.13	−0.05	0.961	0.995	*Sex*	−1.37	0.63	−2.18	0.032	0.066
	*Time*	−2.64	0.46	−5.68	< 0.001	**< 0.001**	*Time*	−0.59	0.10	−6.05	< 0.001	**< 0.001**
	Sex*Time	−0.31	0.66	−0.47	0.639	0.741	Sex*Time	0.49	0.14	3.54	< 0.001	**0.002**
**Active Time (s)**	*Intercept*	285.6	3.03	94.22	< 0.001	---	*Intercept*	274.24	2.35	116.49	< 0.001	---
	*Sex*	4.25	4.29	0.99	0.324	0.469	*Sex*	−10.16	3.33	−3.05	0.003	**0.008**
	*Time*	−5.41	1.81	−2.98	0.004	**0.009**	*Time*	−0.23	0.42	−0.55	0.583	0.726
	Sex*Time	−6.36	2.57	−2.48	0.015	**0.034**	Sex*Time	0.74	0.60	1.24	0.217	0.350
**Average Speed**	*Intercept*	4.79	0.16	30.31	< 0.001	---	*Intercept*	4.02	0.09	42.82	< 0.001	---
	*Sex*	−0.03	0.22	−0.14	0.890	0.956	*Sex*	−0.16	0.13	−1.21	0.230	0.351
	*Time*	−0.46	0.09	−5.01	< 0.001	**< 0.001**	*Time*	−0.11	0.02	−5.58	< 0.001	**< 0.001**
	Sex*Time	0.00	0.13	−0.01	0.995	0.995	Sex*Time	0.10	0.03	3.35	0.001	**0.004**
**Max Speed (m/min)**	*Intercept*	14.13	0.36	39.16	< 0.001	---	*Intercept*	13.23	0.25	52.16	< 0.001	---
	*Sex*	−0.31	0.51	−0.61	0.544	0.717	*Sex*	−0.45	0.36	−1.25	0.216	0.350
	*Time*	−0.65	0.21	−3.05	0.003	**0.008**	*Time*	−0.27	0.05	−5.17	< 0.001	**< 0.001**
	Sex*Time	0.16	0.30	0.53	0.601	0.726	Sex*Time	0.29	0.07	3.83	< 0.001	**0.001**

Open field data, measured over 30-minutes was broken down into 5-minute increments. Each activity measure then had two regressions performed: the first analyzing sex difference from 0–10 minutes and the second from 10–30 minutes. Each model included a term for sex, time, and sex*time interaction. * SE, Standard Error; m, meters; min, minute; q, Benjamini-Hochberg multiple comparisons correction.

## Data Availability

The data sets supporting the conclusion are available from the corresponding author upon request.

## Highlights

- In voluntary wheel running there are large differences between sexes with females demonstrating running a greater distance, longer total time and longer bouts.
- In forced treadmill running at high intensity there were small differences in total time run with females running longer than males.
- In the open field there were small differences in total activity time with females less activity than males.
- Sex differences were dependent on the assay with largest differences found for voluntary running. In general, male mice demonstrate comparable activity intensity to female mice across assays, while females show longer duration of activity especially for volitional tasks.
- Researchers utilizing these assays should account for sex differences as they could potentially mask true findings in an experiment.
